# The impact of body posture on intrinsic brain activity: The role of beta power at rest

**DOI:** 10.1371/journal.pone.0218977

**Published:** 2020-01-24

**Authors:** Brunella Donno, Daniele Migliorati, Filippo Zappasodi, Mauro Gianni Perrucci, Marcello Costantini

**Affiliations:** 1 Department of Neuroscience, Imaging and Clinical Sciences, University “G. d’Annunzio” of Chieti, Chieti, Italy; 2 Institute for Advanced Biomedical Technologies (ITAB), University “G. d’Annunzio” of Chieti, Chieti, Italy; 3 Center for Biomedical Brain Imaging, University of Delaware, Newark, Delaware, United States of America; 4 Department of Psychological, Health, and Territorial Sciences, 'G. d'Annunzio” University of Chieti-Pescara, Italy; Universita degli Studi di Trento, ITALY

## Abstract

Tying the hands behind the back has detrimental effects on sensorimotor perceptual tasks. Here we provide evidence that beta band oscillatory activity in a resting state condition might play a crucial role in such detrimental effects. EEG activity at rest was measured from thirty young participants (mean age = 24.03) in two different body posture conditions. In one condition participants were required to keep their hands freely resting on the table. In the other condition, participants’ hands were tied behind their back. Increased beta power was observed in the left inferior frontal gyrus during the tied hands condition compared to the free hands condition. A control experiment ruled out alternative explanations for observed change in beta power, including muscle tension. Our findings provide new insights on how body postural manipulations impact on perceptual tasks and brain activity.

## Introduction

It is well known that the physical body plays a key role in the way in which the brain encodes the environment; in fact, in everyday life cognitive processes are influenced by the sensory and motor experiences of the body. This idea, which stems from the broader theoretical framework of Embodied Cognition [1], claims that many aspects of cognition are shaped by features of the body [2]. As a consequence, if cognition derives from bodily experiences, the individuals’ physical features might play a critical role in shaping it [3]. A clear example is provided by studies on mental rotation of body parts [4–7], in which participants have to judge the laterality of pictures representing hands and feet while standing in two different postural conditions. In one condition, the subjects’ right hand is placed on the right knee and the left hand behind the back; in the other one, the hand position is reversed. For right-handed subjects, response times increase when participants judge images representing the right hands keeping their right hand behind the back. This effect is not present for images of the left hand, nor for images of the feet. Other studies show analogous results, highlighting an interference of hand posture on the ability to perform mental rotations of hand images [8]. Similarly, subjects’ body orientation has been shown to affect perception of both static and moving objects [9]. All these results suggest that information regarding the current positioning of body or body parts is required for the encoding of visual information. Furthermore, it has been showed that body posture affects the way in which autobiographical memories are accessed and retained by both younger and older adults [10]. Specifically, response times decrease when body position during prompted retrieval of autobiographical events is congruent or similar to the body position in the original events than when body position is incongruent [10]. Nevertheless, the impact of body posture on visual encoding of actions is still under debate. A feasible way to understand how body posture shapes visual encoding of actions is looking at the interaction between posture manipulations and intrinsic brain activity. Specifically, intrinsic brain activity is spontaneously generated by the brain and is not organized in a casual way [11]. The interaction between intrinsic brain activity and posture manipulations is better explained by a study of Thibault and collegues [12]. In this study, prominent alterations of intrinsic brain activity over occipital and frontal regions were induced through orthostatic manipulations. Specifically, an increase of beta and gamma activity was observed while participants lied supine compared to the condition in which they either stand or sat inclined at 45 degrees. Moreover, there is converging evidence showing that intrinsic brain activity plays a key role in perceptual processes [13] involving high-frequency bands (i.e. beta and gamma) [14–16]. For instance, during tasks requiring mental simulation of actions it has been observed a decrease of oscillatory beta power over the sensorimotor regions [17]. Such a decrease reflects the engagement of the motor system corresponding to the disinhibition of motor areas [18]. Conversely, an increased beta power has been shown to reflect inhibition mechanisms related to perceptual and motor systems [19–22]. It is therefore conceivable that postural manipulations may impact visual perception by altering beta-band oscillations. Drawing from this, we investigated the effects of postural manipulations on the intrinsic brain activity, focusing on the beta frequency band. EEG activity was measured in a resting state condition from thirty healthy participants in two different body posture conditions. In one condition, participants were required to keep their hands freely resting on the table. In the other condition, participants were required to keep hands tied behind their back. Moreover, we conducted an EEG-EMG control experiment in order to rule out the presence of confounding variable (i.e. muscle tension). Specifically, subjects were asked to contract and to keep the contraction of specific muscles during the tied hands condition.

## Materials and method

### Participants

Thirty right-handed healthy participants (12 males, mean age = 24.03; SD = 3.2; range = 20–33) were recruited to take part in the study from the student pool. The participants took part in the experiment at ITAB (Institute for Advanced Biomedical Technologies) in Chieti. Participants did not have any personal or close family history of neurological or psychiatric disorders, any brain surgery and any active medication, as self-reported. The study was approved by the local ethics committee of the province of Chieti-Pescara (ID07022013). Participants gave their informed consent before taking part in the study. The study was conducted in accordance with the ethical standards of the 1964 Declaration of Helsinki.

We calculated the power achieved in our study a posteriori. The achieved power was 87% with an α value = 0.05, two tails test.

### Procedure

#### EEG resting-state recording and pre-processing

All participants went through two different conditions (within-subject design): i) EEG resting-state when their hands were free (free condition) and ii) EEG resting-state when their hands were tied behind the back (tied condition). The two conditions were randomized across subjects. In the two blocks, participants had to keep their eyes open and fixate a cross in front of them placed on the computer screen. EEG activity was measured at rest for 4 minutes.

We used a 64 electrodes cap (model BrainCap, BrainAmp MR Plus amplifier, Brain Products), placed according to the 10–20 International System. We used 2 electro-oculographic channels on the right and left temple to monitor eye movement and for off-line artefact rejection. The reference electrode was positioned in correspondence of FCz electrode while the ground electrode was placed in the Inion (Iz).

The impedance was measured before each recording and was kept below 10 kΩ. All the data were processed using EEGLAB software implemented in MATLAB [23].

We acquired online data at 5 kHz, band-pass filtered from 0.016 to 250 Hz. Data were off-line filtered between 1 Hz (high-pass filtering) to 30 Hz (low-pass filtering) and downsampled at 250 Hz for the current analysis. We detected and removed bad channels using a threshold with a probability at 5% (*pop rejchan*). Subsequently, we rejected the continuous data using a threshold with a probability at 10% (*pop rejcont*).

Finally, we computed the Independent Component Analysis, using the FastICA algorithm [24] to identify and reject manually noise, ocular, cardiac, muscular artefacts and bad channels. At this point, we interpolated rejected channel and EEG signal was re-referenced to the common average reference.

To exclude that differences in the beta band power in the tied hands with respect to free hands condition is not a consequence of muscular tension during the condition of tied hands, a control experiment was done. We co-registered EEG-EMG resting-state activity in 4 subjects (3 males, mean age = 26.75; SD = 3.6; range = 24–32) during a low-level isometric contraction of the muscles for 4 minutes, recorded along with the EMG. During the tied hands condition, we asked to participants to contract deltoids, triceps, pectorals and dorsal muscles and to keep the contraction for 4 minutes as stable as possible.

Specifically, we recorded the contraction through 8 electromyographic channels: right and left deltoid, right and left pectoral, right and left dorsal, right and left triceps. A 32 electrodes cap (model BrainCap, BrainAmp MR Plus amplifier, Brain Products) was used. As the aim of the recording was to check the muscular artefact topography over the scalp, only ocular, cardiac and movement artefacts was rejected by ICA procedure. We computed the power spectrum density only for the beta band. The aim was to confirm a qualitative difference between the beta power spectrum scalp topography of the difference between tied versus free hands condition, obtained in the main experiment and the difference between the beta power scalp topography of the difference between contraction (tied hands condition) versus free hands condition, obtained in the control experiment. For the control experiment, for each subject we performed the beta power spectrum scalp topography of the difference between the two conditions.

#### EEG cleaning: Criteria of exclusion

In order to rule out the presence of confounding variables (i.e. muscular artefacts) and to better identify them from neural activity per sé, we applied high-pass filtering (1 Hz) EEG signals before applying FastICA [24]. It is well known that such artefacts are typically caused by muscle activity near the head, such as head movements and are characterized by high-frequency activity (> 20 Hz) [25].

It is well known that high-pass filtering EEG signals before applying ICA may improve the quality of the artefacts separation. In fact, this procedure represents a standard procedure to remove drifts prior to ICA-based artefact removal and the benefit has been demonstrated in several studies [26]. This standard procedure allowed us to isolate the EEG signal from the EMG and finally to compare the topographies of the EEG signal of the main experiment with those of the control experiment.

### EEG data analysis

#### Main experiment

We computed the power spectrum density for all electrodes using the periodogram Welch procedure (Hamming windowing function; window length 4 seconds; no overlap). The four classical EEG frequency bands were considered (delta: 1–4 Hz, theta: 4–8 Hz, alpha: 8–13 Hz and beta: 13–30 Hz). Delta, theta and alpha bands were used to control that the difference tied versus free hands condition was specific for the beta band.

Then we extracted the power of delta, theta, alpha and beta bands calculating the mean values of the power spectrum for all frequency bands and for the two conditions (free and tied) described above. The mean values were transformed into decibel scale (10 × log10[μV^2^]) in order to normalize the data [27].

To establish whether there were significant differences in power for all frequency bands between two conditions, a non-parametric cluster-based permutation test was performed using FieldTrip toolbox in MATLAB [28]. To investigate cortical generators of electrophysiological oscillations, we computed signal source analyses on the basis of the results obtained at the scalp level.

The exact low resolution brain electromagnetic tomography (eLORETA) method in frequency domain was used to compute the cortical three-dimensional distribution of current density [29]. Computations are made in a realistic head model [30] using the Montreal Neurological Institute (MNI) Colin27 T2 template obtained from BrainWeb, (http://www.bic.mni.mcgill.ca/brainweb/).

Starting from estimated cortical distribution of generators of beta electrophysiological oscillations, the analysis of differences between free and tied hands condition showed a specific modulation whose significant neural source is localized in a specific cortical area.

### EEG results

#### Main experiment

To test our hypothesis, we performed a non-parametric cluster-based permutation test for the power of all frequency bands between the two conditions (free and tied).

For each sample, a dependent-sample *t*-value was computed. All samples were selected for which this *t*-value exceeded an a priori threshold (uncorrected *p* < 0.05) and these were subsequently spatially clustered. The sum of the *t* values within a cluster was used as the cluster-level statistic. A reference distribution of cluster *t*-values was obtained by randomization of data across the two conditions for 5000 times and was used to evaluate the statistic of the actual data.

**Fig 1:**
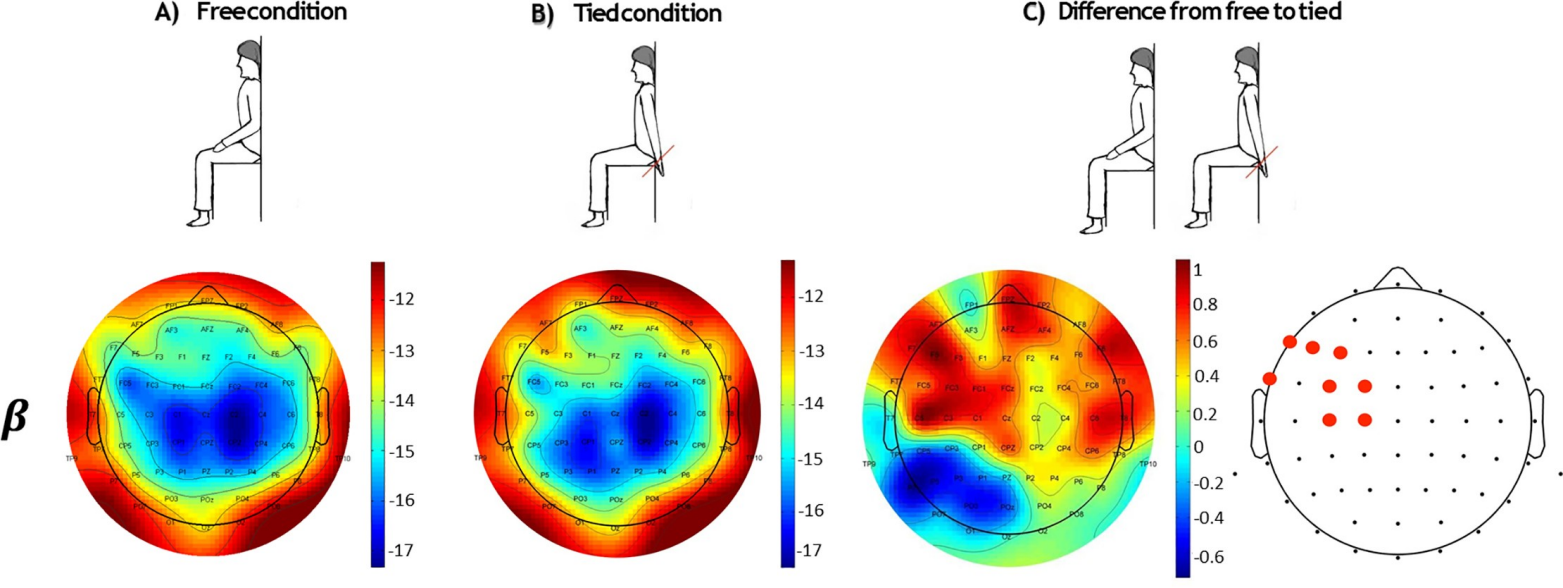
Scalp distributions of average beta power (decibel: 10 × log10[μV^2^]) in the free condition (Panel A) and tied condition (Panel B). Panel C represents the differences in beta power between the two conditions. Red dots represent electrodes in the significant cluster (*ps* = 0.02).

The non-parametric cluster-based permutation test revealed a significant difference between free and tied condition only in the beta band power (*ps* = 0.02). The analysis revealed increased beta power in tied hands condition compared to free hands condition and the difference between these two conditions was most pronounced over left inferior frontal electrodes (Table 1 and Fig 1).

**Table 1:** Fronto-central electrodes in the significant cluster in beta power band (decibel: 10 × log10[μV^2^]) in the two experimental conditions (*ps* = 0.02).

	Free Condition		Tied Condition	
Electrodes	Mean	SD	Mean	SD
**F3** **C3** **F7** **FC1** **C1** **FC3** **F5** **FT7**	-14,44 -15,89 -14,50 -15,30 -16,72 -15,58 -14,18 -13,74	3,16 2,94 2,72 3,26 3,07 3,13 2,57 2,86	-13,63 -15,04 -13,60 -14,48 -15,95 -14,68 -13,13 -13,15	4,25 3,96 3,87 3,86 3,75 3,68 3,30 3,28

The non-parametric cluster-based permutation test did not reveal any significant differences between free and tied condition in the other frequency bands (all *ps* > 0.05).

**Fig 2:**
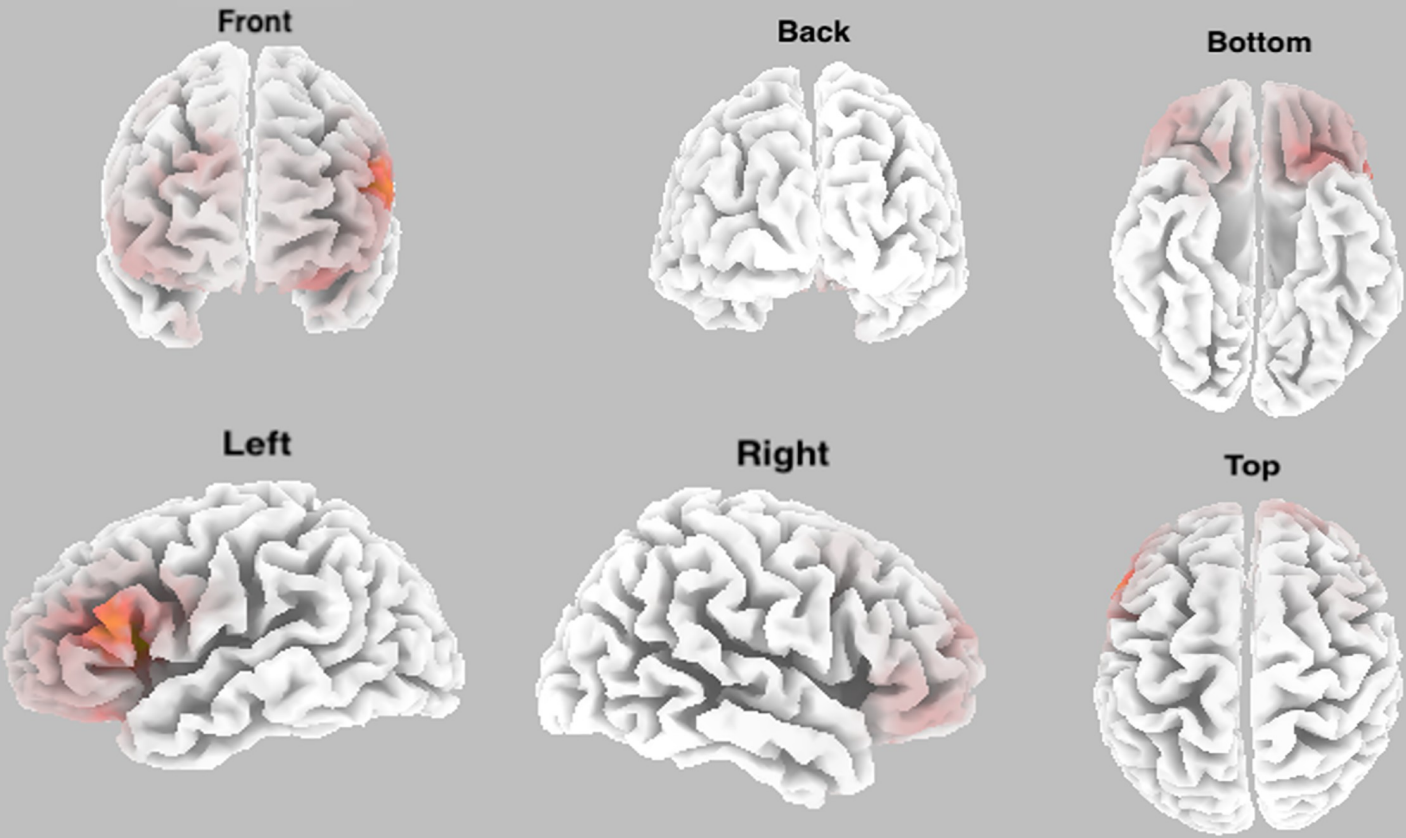
Source localization for beta power spectral data between free hands and tied hands conditions: eLORETA best match. Three-dimensional model reconstruction.

As regards signal source localization, the comparison between electrophysiological activity for beta power between free and tied hands conditions showed that the main signal source was in the left inferior frontal gyrus (l-IFG) (MNI: *x* = -35, *y* = 10, *z* = 15; *t* = 7.21) (Fig 2).

#### Control experiment

To exclude that differences in the beta band power in the tied hands with respect to free hands condition is not a consequence of muscular tension during the condition of tied hands, we compared the scalp distributions of average beta power relating to the difference between tied versus free hands condition, obtained in the main experiment and the scalp distributions of average beta power relating to the difference between contraction (tied hands condition) versus free hands condition, obtained in the control experiment. For each subject, we performed the beta power spectrum scalp topography of the difference between contraction (tied hands condition) versus free hands condition.

**Fig 3:**
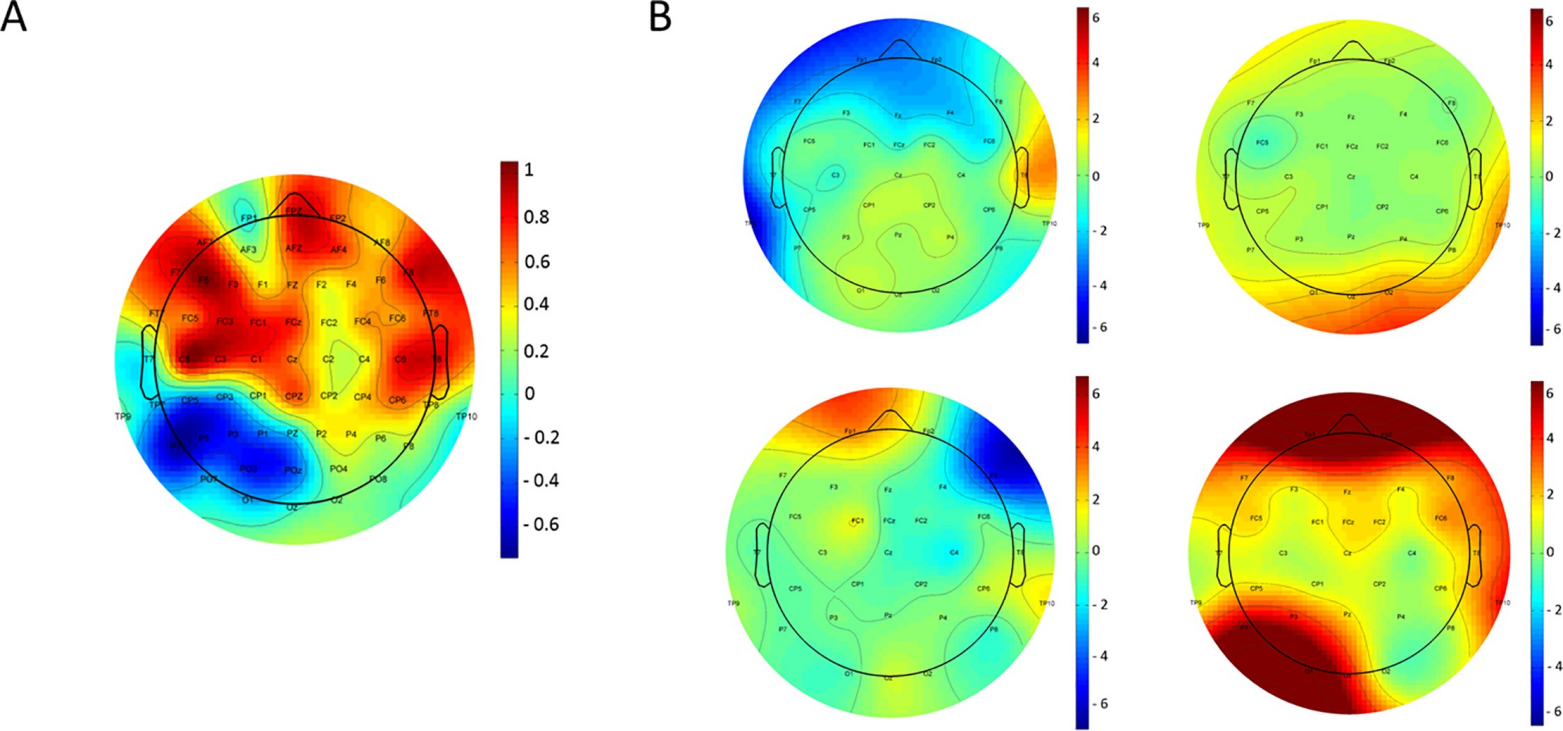
Scalp distributions of average beta power (decibel: 10 × log10[μV^2^]) of difference between tied versus free condition (Panel A) and 4 scalp distributions of average beta power (decibel: 10 × log10[μV^2^]) of difference between contraction (tied hands condition) versus free condition for each subject (Panel B).

From each scalp topography, the maximum of the muscular artefact was located in the temporal, fronto-polar and parietal regions. In particular, muscular contamination was not present over the EEG channels where a significant difference between tied and free hands condition was found in the main experiment (F3, C3, F7, FC1, C1, FC3, F5, FT7) (Fig 3).

Moreover, a similar topography was not found by visual inspection of beta band topographies of single subjects before the ICA algorithm application for artefact removal.

## Discussion

Our physical body plays a key role in the way in which the brain encodes stimuli from the environment; in fact, in everyday life cognitive processes are deeply influenced by the sensory and motor experiences of our body. This theory of cognition, known under the general topic of Embodied Cognition [1], claims that many aspects of cognition are shaped by body features [2]. This theoretical framework implies that if knowledge is obtained through bodily experiences, it is constrained not only by the experiences and situations encountered, but also by the physical features of the individuals [3]. In the present study, resting state EEG activity was measured from thirty healthy participants in two different body posture conditions. In the free hands condition, participants were required to keep their hands freely resting on the table; in the tied hands condition, participants were required to keep hands tied behind their back. Power spectrum analysis revealed an increased beta power in the tied hands condition compared to the free hands condition. This difference was most pronounced over left frontal electrodes.

The way in which body posture manipulations modulate sensorimotor perceptual tasks is well known, as well as the involvement of beta power in the inhibition and disinhibition of motor mechanisms; however, the impact of the body posture on beta band oscillatory activity in a resting state condition is still unknown. Our results suggest that an increased beta power in the tied hands condition, compared to free hands condition, which might also explain the constrained hands effect commonly observed when participants perform perceptual action-related tasks.

However, does beta power activity in sensorimotor regions effectively play a role in processing such stimuli? And to which extent is it involved in their processing? Previous studies have shown that observation of graspable objects, which is known to recruit sensorimotor resources [30] and to be affected by postural manipulations, is associated with a decrease in beta band power. Similarly, suppression of oscillatory activity within the mu (8–13 Hz) and beta (13–30 Hz) frequency bands over sensorimotor regions has been associated with action execution, as well as action observation [31–35]. Moreover, it has been found that also passive observation of manipulatable objects elicits neural responses similar to the ones elicited during passive observation of others’ actions [36–39].

Hence, increasing evidence suggests that the power of beta rhythm typically decreases during the preparation and the execution of a movement [40]; it increases in the motor cortex during active immobilization [41], postural maintenance [42], proactive inhibition [43], as well as when a movement have to be withheld or voluntary suppressed [44], but also before an expected postural challenge [45]. Moreover, strong pieces of evidence have demonstrated that beta power enhancement with transcranial alternating cortical stimulation has been shown to induce motor inhibition [46].

Similar results have been found when rhythmic activity is induced in the motor cortex of healthy participants using transcranial current stimulation. Specifically, the stimulation in the beta band frequency range, reflecting an increased beta power, is particularly effective in slowing movements and increasing the threshold of inducing a motor response [47–49].

Moreover, the role played by the beta band in inhibition/disinhibition of neural motor system is supported also by studies on clinical populations. Specifically, the functional relevance of the beta band rhythm in the disinhibition of neuronal populations becomes particularly clear in Parkinson’s disease (PD), where pathological high beta band activity severely compromises movement initiation and execution [50, 51]. Taken together, these findings support the idea that the beta band power maintains the functioning of the sensorimotor cortex. Our data also support the hypothesis that beta band activity may signal the tendency of the sensorimotor system to maintain the status quo [52]. An interesting hypothesis is that beta band activity may allow the more efficient processing of feedback (e.g. proprioceptive signals) which is required for monitoring the status quo and recalibrating the sensorimotor system [52].

Furthermore, in order to investigate cortical generators of electrophysiological oscillations of beta frequency band, we performed signal source localization for beta power spectral data. The comparison between electrophysiological activity in the free hands and tied hands condition showed that the main signal source was localised in the left inferior frontal gyrus (l-IFG). The involvement of the l-IFG in processing action related stimuli has been shown by a large amount of studies. For instance, it has been shown that this cortex is critically involved not only in planning and executing object-related hand actions [53, 54], but also in processing both others’ object-related actions and action-related features of objects. Moreover, a large number of studies have demonstrated that viewing another's object related action recruits the left ventral premotor cortex (PMv) as if the viewer were performing that action herself [55–60]. Finally, this area has been shown to be involved in response inhibition in a Go/NoGo task, demonstrating how the integrity of this area is critical for successful implementation of inhibitory control over motor responses [61], as well as it is crucially involved in processing visual features of objects in terms of the actions they might afford [62–65]. In this context, it has been demonstrated how l-IFG and PMv are significantly activated during gesture planning and tool use actions [66].

Furthermore, regardless of the origin of the observed effect (muscle tension during the tying hands), we have shown how the neural outcome, namely the increase in beta power in the tied hands with respect to free hands condition, cannot be considered as a consequence of muscular tension during the condition of tied hands. In this regard, we compared the scalp topography of beta power distribution of the control experiment and the main experiment. To confirm that the data obtained were not caused by muscular tension, we compared the scalp topography of beta power distribution relating to the difference between contraction (tied hands condition) versus free hands condition for each subject obtained during the control experiment and the topography of the average beta power distribution relating to the difference between tied versus free hands condition obtained during the main experiment.

To this aim, we conducted an EEG-EMG control experiment. The two conditions were the same as the main experiment (free and tied hands). Specifically, during the tied hands condition we asked participants to contract deltoids, triceps, pectorals and dorsal muscles and to keep the contraction as stable as possible. EEG activity in a resting state condition and EMG muscle activity in a resting state condition were measured from four participants. Results showed that muscular contamination was not present over the EEG channels where a significant difference between tied and free hands condition was found in the main experiment. To sum up, we have shown the effect of tying the hands on intrinsic brain activity and how this manipulation can change the activity in the beta frequency band in a resting state condition. Our result might contribute to explain the constrained hand effect commonly observed when participants perform perceptual action-related tasks.

## Limitations and future perspectives

In the control experiment, a main limitation could be represented by the small-size of sample which could make the results inaccurate because the data collected is not enough: in fact, we recruited only 4 participants for EMG-EEG study. Indeed, it would be appropriate to increase the sample size of the EMG-EEG study to make the two samples of the main experiment and of the control experiment comparable.
